# Predictive value of radiomics modeling based on dynamic and static ^18^F-FDG PET/CT imaging for the differential diagnosis of lymph nodes in lung cancer

**DOI:** 10.3389/fonc.2025.1637366

**Published:** 2025-09-22

**Authors:** Xieraili Wumener, Zhiheng Hu, Jiuhui Zhao, Hongjian Wang, Yarong Zhang, Yaohong Deng, Jun Zhao, Ying Liang

**Affiliations:** ^1^ Department of Graduate School, Dalian Medical University, Dalian, China; ^2^ Department of Nuclear Medicine, National Cancer Center/National Clinical Research Center for Cancer/Cancer Hospital & Shenzhen Hospital, Chinese Academy of Medical Sciences and Peking Union Medical College/Shenzhen Clinical Research Center for Cancer, Shenzhen, China; ^3^ Department of Nuclear Medicine, The Fourth Affiliated Hospital of Xinjiang Medical University, (Xinjiang Uygur Autonomous Region Hospital of Traditional Chinese Medicine), Urumqi, China; ^4^ Shanghai United Imaging Intelligence Co., Ltd, Shanghai, China; ^5^ Department of Nuclear Medicine, Shanghai East Hospital Tongji University, Shanghai, China

**Keywords:** lung cancer, ^18^F-FDG, PET/CT, dynamic, radiomics model

## Abstract

**Background:**

We aimed to identify the most effective machine learning model for predicting the differential diagnosis of lymph nodes (LNs) in lung cancer using dynamic and static ^18^F-fluorodeoxyglucose (FDG) positron emission tomography/computed tomography (PET/CT) imaging.

**Methods:**

A total of 279 pathologically confirmed LNs from 74 patients with lung cancer were retrospectively analyzed. These were randomly divided into a training group (*n* = 196) and a test group (*n* = 83) at a ratio of 7:3. The radiomics features of the images were extracted from CT, dynamic PET (dPET), and static PET (sPET) images and were screened for the most predictive value. Support vector machine (SVM), logistic regression (LR), and random forest (RF) machine learning models were built using the optimal radiomics features. The best quantitative prediction model was suggested using SUV_max_ and *K*_i_ based on LNs. A composite model was built combining the best machine learning model and the quantitative model. Receiver operating characteristic (ROC) curves were used to evaluate the predictive ability of the machine learning, quantitative, and composite models for LN metastasis in lung cancer.

**Results:**

Of the three machine learning models, the RF model demonstrated the greatest predictive efficacy in both the training [area under the curve (AUC) = 0.823] and test groups (AUC = 0.819). The quantitative model based on *K*_i_ showed good predictive efficacy in both the training (AUC = 0.772) and test groups (AUC = 0.805). A composite model based on both the RF machine learning model and the quantitative model demonstrated superior predictive efficacy. The AUCs in the training and test groups were 0.844 and 0.835, respectively. Decision curve analysis showed that the composite model had better net benefit and clinical value.

**Conclusion:**

A composite model based on an RF model of PET/CT+K_i_ images combined with dynamic quantitative *K*_i_ is highly effective in differentiating FDG-avid LN metastasis in lung cancer. This model provides greater net benefit and clinical value.

## Introduction

Lung cancer is the leading cause of both morbidity and mortality (1). Accurate N staging is essential for individualized treatment planning and prognosis in lung cancer (2). Patients diagnosed with stage N3 not only lose the chance of undergoing surgery, but the 5-year survival also drops to 6% (3). Consequently, improving the accuracy of the lung cancer N staging is one of the current clinical concerns.

^18^F-fluorodeoxyglucose (FDG) positron emission tomography/computed tomography (PET/CT) is commonly used for lung cancer staging (4). A previous meta-analysis (5) showed FDG PET/CT for the mediastinal staging of patients with non-small cell lung cancer (NSCLC) to have a sensitivity of 0.81 (0.70–0.89) and a specificity of 0.79 (0.70–0.87). The semi-quantitative metabolic parameter known as standard uptake value (SUV_max_) is affected by various factors, which reduces the specificity of FDG PET/CT for N staging. The presence of lung cancer alongside infectious lung diseases such as tuberculosis, infection, and granulomatous inflammation, in particular, reduces the specificity of FDG PET/CT for precise staging by approximately 16%–25% (6–8).

Dynamic PET (dPET) involves the continuous acquisition of imaging data over a period of time. The extracted fully quantitative metabolic parameters (e.g., *K*_i_) provide a more accurate characterization of the different metabolic phases of FDG, thereby reflecting the pathophysiological mechanisms of the disease (9–11). In recent years, the clinical application of dPET in tumor diagnosis and treatment has also become a popular area of research. We have previously carried out a study on the clinical value of dPET in lung cancer (12–15). It was concluded that dPET has good value in the differential diagnosis, N staging, and prediction of the epidermal growth factor receptor (EGFR) status in lung cancer; in particular, the *K*_i_ can improve specificity (12–15). The results of the lung cancer N-staging study concluded that, compared with SUV_max_, there is good specificity in the differential diagnosis of FDG-avid lymph nodes (LNs) when the *K*_i_ cutoff value is 0.022 ml g^−1^ min^−1^ (0.918 *vs*. 0.388) (15). A validation study has also shown that, when the SUV_max_ and *K*_i_ are used in combination for diagnosis, the diagnostic efficacy is further improved (12). Therefore, dynamic metabolic parameters are expected to reliably indicate the N stage of lung cancer.

To our knowledge, there are no studies reporting on the predictive value of radiomic features based on dPET for the N staging of lung cancer. In this study, we investigated the predictive value of radiomics models, quantitative models, and combined models based on dPET and FDG PET/CT images for the differential diagnosis of FDG-avid LNs in lung cancer.

## Materials and methods

### Patients

The study was approved by the Ethics Committee of X Hospital (KYLH2022-1). Written informed consent was obtained from all patients before dPET and FDG PET/CT imaging.

A total of 323 patients underwent dPET (chest, 65 min) and static FDG PET/CT (sPET/CT) imaging (whole body, 10–20 min) from May 2021 to December 2024. All patients had lung nodules or masses identified on a chest CT scan, and none of the patients received anti-infective or antitumor therapy prior to undergoing a dPET+sPET/CT scan. Of these patients, 261 had lung cancer confirmed by puncture and/or surgical pathology.

We retrospectively collected 279 FDG-avid LNs from 74 patients with pathologically confirmed lung cancer. The 74 patients were selected from 261 lung cancer patients. On the sPET/CT scan, mediastinal or pulmonary hilar region LNs were considered FDG-avid LNs if their FDG uptake exceeded the mediastinal blood pool. All 279 FDG-avid LNs were confirmed by pathology, and the LNs were included according to their distribution and size on the sPET/CT scan after a one-to-one correspondence with the pathological findings. The locations of the LNs according to the International Association for the Study of Lung Cancer (IASLC) are shown on the LN map (16). The time interval between the dPET+sPET/CT scan and receipt of the pathology results was less than 2 weeks.

We collected the dPET+sPET/CT scans and the clinical features of FDG-avid LNs. The dPET+sPET/CT scan features included the primary focus site, the primary focus SUV_max_, the FDG-avid LN zoning, the LN short and long diameters, the LN-SUV_max_, and the LN-*K*_i_. The clinical characteristics included gender, age, primary lung cancer pathology, and LN pathology.

Based on the pathological findings, of the 279 FDG-avid LNs, 161 (57.71%) were metastatic and 118 (42.29%) were non-metastatic. The participants were randomly divided into two groups: a training group (*n* = 196) and a test group (*n* = 83).

### dPET and sPET/CT data acquisition and image reconstruction

**Figure 1:**
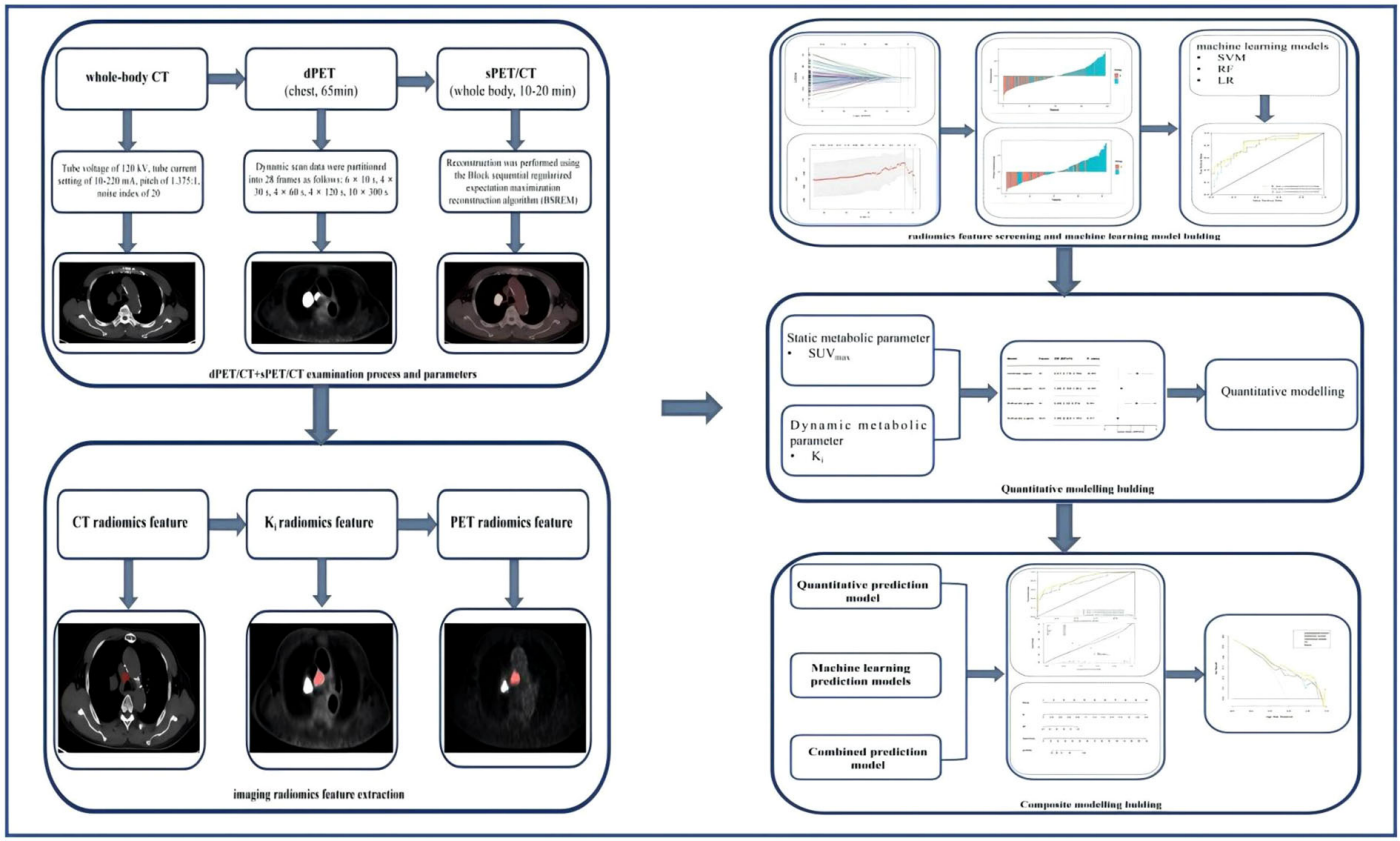
Dynamic positron emission tomography (dPET) and static PET (sPET) acquisition process and model screening and establishment in each group.

Both the dPET and sPET/CT scans were performed using a Discovery MI PET/CT (GE Healthcare, Milwaukee, WI, USA). **Figure 1** illustrates the dPET and sPET/CT examination processes, including data acquisition, image reconstruction, and metabolic parameter acquisition.

Dynamic *K*_i_ images and quantitative metabolic values were obtained based on a two-tissue irreversible compartment model. In this model, it was assumed that ^18^F-FDG was taken up unidirectionally (i.e., *k*_4_ = 0) and was irreversibly trapped in tissue as ^18^F-FDG-6-PO (17). The image-derived input function (IDIF) was extracted from the ascending aorta by drawing a region of interest (ROI) with a diameter of 10 mm on six consecutive slices in an image obtained by combining early time frames (0–60 s), in which the effects of motion and partial volume are less pronounced than that in the left ventricle. Two experienced nuclear medicine physicians used the ITK-snap software (version 4.9) to display the 3D volume of interest (VOI) for each LN in the *K*_i_ images and to calculate the quantitative values.

Two experienced nuclear medicine physicians independently reviewed the static images. Based on the distribution of the LNs in the puncture and/or pathological findings, the LN long and short diameters were measured on 5-mm CT scans according to the one-to-one correspondence principle, and the LN site and LN-SUV_max_ were recorded on the sPET/CT scan.

### Pathological evaluation

The diagnosis was based on two factors: the appearance under the microscope and the immunohistochemical results. Two experienced pathologists made the diagnosis independently.

### Radiomics feature extraction

**Figure 1** illustrates the radiomics feature extraction process. All of the patients’ 2.79-mm PET, 3.75-mm chest CT, and 2.79-mm *K*_i_ images were exported to DICOME from the PET/CT workstation. The DICOM format files were then imported into the radiomics version of the uAI Research Portal (version 3.0.1; https://pyradiomics.readthedocs.io/en/) to create outlines and to extract the radiomics features. A junior physician performed manual delineation of the VOI layer-by-layer on the PET (SUV threshold of 40%), CT, and *K*_i_ images in a blinded fashion using a software annotation tool. The results outlined by the VOI were then reviewed by another senior doctor.

Prior to the radiomics feature extraction, the distribution of the image voxels in all segmented VOIs was standardized using mean normalization. A total of 4,362 radiomics features were extracted based on the CT, PET, and *K*_i_ images, 1,454 of which were CT features, 1,454 were PET features, and 1,454 were *K*_i_ features. These radiomics features included: first-order statistics and shape features, gray-level co-occurrence matrix (GLCM) features, gray-level run length matrix (GLRLM) features, gray-level size zone matrix (GLSZM) features, neighboring gray tone difference matrix (NGTDM) features, and gray-level dependence matrix (GLDM) features. Advanced features were achieved using five filters: original, Laplacian of Gaussian (LoG), mean, box mean, and additive Gaussian noise. The parameters were as follows: for original, native image intensities were used without any spatial filtering; for LoG, edge enhancement was performed using 3D LoG filtering with a Gaussian kernel (*σ* = 3.0 mm); for the mean, uniform mean filtering was applied with a 3 × 3 × 3 voxel smoothing kernel; for the box mean, cubic mean filtering was implemented using a 5 × 5 × 5 voxel kernel; and for additive Gaussian noise, a zero-mean Gaussian noise (10% of the VOI standard deviation) was introduced to simulate acquisition noise.

### Radiomics feature screening and modeling

**Figure 1** shows the radiomics feature screening and modeling processes. The extracted radiomics features were then put through a process of *Z*-score normalization. This was performed so that any differences in the dimensions of the index could be managed. Subsequently, Student’s *t*-test was used on the training set to compare the features that conformed to a normal distribution in order to distinguish between FDG-avid LN metastasis and non-metastasis. For features that did not follow a normal distribution, the Mann–Whitney *U* test was used for the initial feature selection. Among these features, Pearson’s correlation coefficient was calculated between each feature–label pair that follows a normal distribution, and features with |*r*| > 0.6 were selected. LASSO (least absolute shrinkage and selection operator) logistic regression was used to select the radiomics features and to calculate the radiomics score (Rad-score), which was then iteratively validated using 10-fold cross-validation. Three machine learning models were constructed according to the radiomics features of the images: support vector machine (SVM), random forest (RF) classifier, and logistic regression (LR) models.

### Machine learning modeling, quantitative modeling, and composite model building and assessment

For the construction of the quantitative model, one-way logistic regression analyses were first performed for SUV_max_ and *K*_i_. The correlated features were then further incorporated into the multifactor logistic regression to determine the risk predictors. For the construction of the PET/CT+*K*_i_ machine learning model, after comparing the efficacy of three machine learning models (i.e., RF, SVM, and LR), the machine learning model with the best overall prediction efficacy was selected to obtain the PET/CT+*K*_i_ machine learning model. For the construction of the composite model, a PET/CT+*K*_i_+quantitative composite model was obtained by applying logistic regression analysis to the PET/CT+K_i_+quantitative composite model after averaging the weights of the predicted values of the PET/CT+K_i_ optimal machine learning model and the quantitative model. **Figure 1** shows the machine learning modeling, quantitative modeling, and composite model development and evaluation processes.

### Statistical analysis

Statistical analyses and model construction were carried out using the R statistical software package (version 4.1.1) and Python programming language (version 3.7). The groups were compared using the Wilcoxon rank-sum test or the independent-samples *t*-test. Single-factor and LASSO logistic regression were used to determine the significant risk predictors and radiomics features, as well as the calibration curves. The predictive ability of the model was assessed using receiver operating characteristic (ROC) curves, the area under the curve (AUC), and calibration curves. DeLong’s test was used to determine whether the difference in the efficacy between the models was statistically significant.

## Results

### Patient and lesion features

The patient and LN features are shown in **Table 1**. This study included a total of 279 LNs from 74 patients with lung cancer, of whom 51 (70.83%) were men and 23 (31.93%) were women, with an average age of 61.8 ± 10.0 years. Of the 279 LNs that were pathologically confirmed, 118 (42.29%) were non-metastatic and 161 (57.71%) were metastatic. The enrolled LNs were randomly divided into a training group and a test group in a 7:3 ratio. There were 196 LNs in the training group and 83 LNs in the test group.

**Table 1:** Patient and lymph node (LN) characteristics.

Clinical characteristic		Training group (*N* = 196)			Test group (*N* = 83)		*p*
		Non-metastatic (*n* = 83)	Metastatic (*n* = 113)	*p*	Non-metastatic (*n* = 35)	Metastatic (*n* = 48)	
Lung cancer primary focus location	RUL	33 (39.8%)	22 (19.5%)	0.007	21 (60.0%)	8 (16.7%)	<0.001
	RML	10 (12.0%)	8 (7.1%)		4 (11.4%)	2 (4.2%)	
	RLL	5 (6.0%)	16 (14.2%)		2 (5.7%)	7 (14.6%)	
	LUL	20 (24.1%)	39 (34.5%)		7 (20.0%)	17 (35.4%)	
	LLL	15 (18.1%)	28 (24.8%)		1 (2.9%)	14 (29.2%)	
LN pathology type	SCC	17 (20.5%)	16 (14.2%)	0.002	11 (31.4%)	4 (8.3%)	0.007
	AC	63 (75.9%)	71 (62.8%)		22 (62.9%)	38 (79.2%)	
	SCLC	2 (2.4%)	15 (13.3%)		–	3 (6.2%)	
	Other	1 (1.2%)	11 (9.74%)		2 (5.71%)	2 (6.25%)	
LN zoning	1	–	1 (0.9%)	–	–	4 (8.33%)	–
	2	6 (7.2%)	3 (2.7%)		1 (2.9%)	1 (2.1%)	
	3A	1 (1.2%)	5 (4.4%)		1 (2.9%)	1 (2.1%)	
	4	4 (4.8%)	14 (12.4%)		13 (37.14%)	16 (33.33%)	
	5	16 (19.3%)	19 (16.8%)		1 (2.9%)	3 (6.2%)	
	6	2 (2.4%)	6 (5.3%)		–	1 (2.1%)	
	7	3 (3.6%)	7 (6.2%)		4 (11.4%)	6 (12.5%)	
	8	9 (10.8%)	20 (17.7%)		–	–	
	9	1 (1.2%)	2 (1.8%)		–	–	
	10	13 (15.66%)	3 (2.66%)		5 (14.29%)	1 (2.1%)	
	11	28 (33.74%)	28 (24.78%)		10 (28.57%)	10 (20.83%)	
	12	–	4 (3.5%)		–	1 (2.1%)	
Long diameter (cm)		1.20 (1.00–1.50)	1.40 (1.00–2.00)	0.008 <0.001	1.30 (1.05–1.40)	1.60 (1.17–2.02)	0.027
Short diameter (cm)		0.80 (0.70–1.00)	1.00 (0.80–1.40)		1.00 (0.80–1.00)	1.10 (1.00–1.40)	0.001
SUV_max_		4.00 (2.80–6.00)	6.70 (4.50–10.00)	<0.001	3.70 (3.00–5.75)	7.00 (4.62–10.65)	<0.001
*K*_i_		0.01 (0.01–0.02)	0.02 (0.01–0.04)	<0.001	0.01 (0.01–0.02)	0.03 (0.02–0.05)	<0.001

RUL, right upper lobe; RML, right middle lobe; RLL, right lower lobe; LUL, left upper lobe; LLL, left lower lobe; SCC, squamous cell carcinoma; AC, adenocarcinoma carcinoma; SCLC, small cell lung cancer.

### Radiomics feature screening

**Figure 2:**
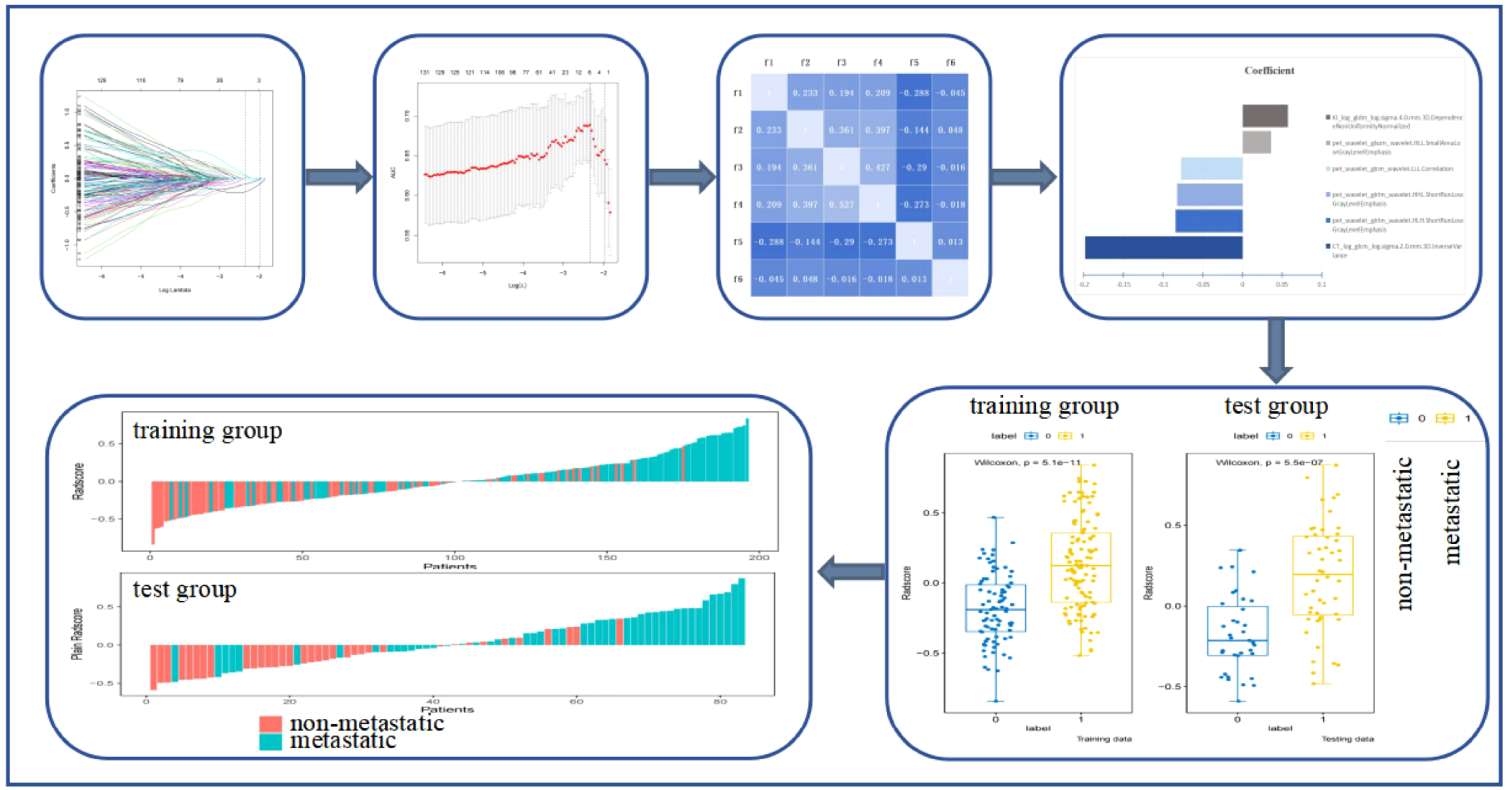
Process and results of the screening for radiomics features. *f1*, CTlog_glcm_log.sigma.2.0.mm.3D.InverseVariance; *f2*, pet_wavelet_glcm_wavelet.LLL.Correlation; *f3*, pet_wavelet_glrlm_wavelet.HLH.ShortRunLowGrayLevelEmphasis; *f4*, pet_wavelet_glrlm_wavelet.HHL.ShortRunLowGrayLevelEmphasis; *f5*, pet_wavelet_glszm_wavelet.HLL.SmallAreaLowGrayLevelEmphasis; *f6*, KI_log_gldm_log.sigma.4.0.mm.3D.DependenceNonUniformityNormalized.

**Figure 2** shows the process and the results of the screening for radiomics features. A total of 4,362 radiomics features were extracted from the CT, PET, and *K*_i_ images: 1,454 from CT, 1,454 from PET, and 1,454 from *K*_i_. A total of 319 radiomics features were selected using Student’s *t*-test or the Mann–Whitney *U* test and Pearson’s correlation analysis. The six most significant radiomics features were subsequently selected using LASSO logistic regression. These included one feature for CT (GLCM), three features for PET (GLRLM, GLCM, and GLSZM), and one feature for *K*_i_ (GLDM). The final PET/CT+*K*_i_ radiomics feature score formula was calculated by summing the coefficients for the retained radiomics features, which were weighted according to their importance.

Rad_score_ = −0.1979*CTlog_glcm_log.sigma.2.0.mm.3D.InverseVariance+-0.0843*pet_wavelet_glrlm_wavelet.HLH.ShortRunLowGrayLevelEmphasis+-0.08257*pet_wavelet_glrlm_wavelet.HHL.ShortRunLowGrayLevelEmphasis+-0.07745*pet_wavelet_glcm_wavelet.LLL.Correlation+0.035966*pet_wavelet_glszm_wavelet.HLL.SmallAreaLowGrayLevelEmphasis+0.057188*KI_log_gldm_log.sigma.4.0.mm.3D.DependenceNonUniformityNormalized.

### Predictive value of the radiomics features in the differential diagnosis of FDG-avid LNs

**Figure 3:**
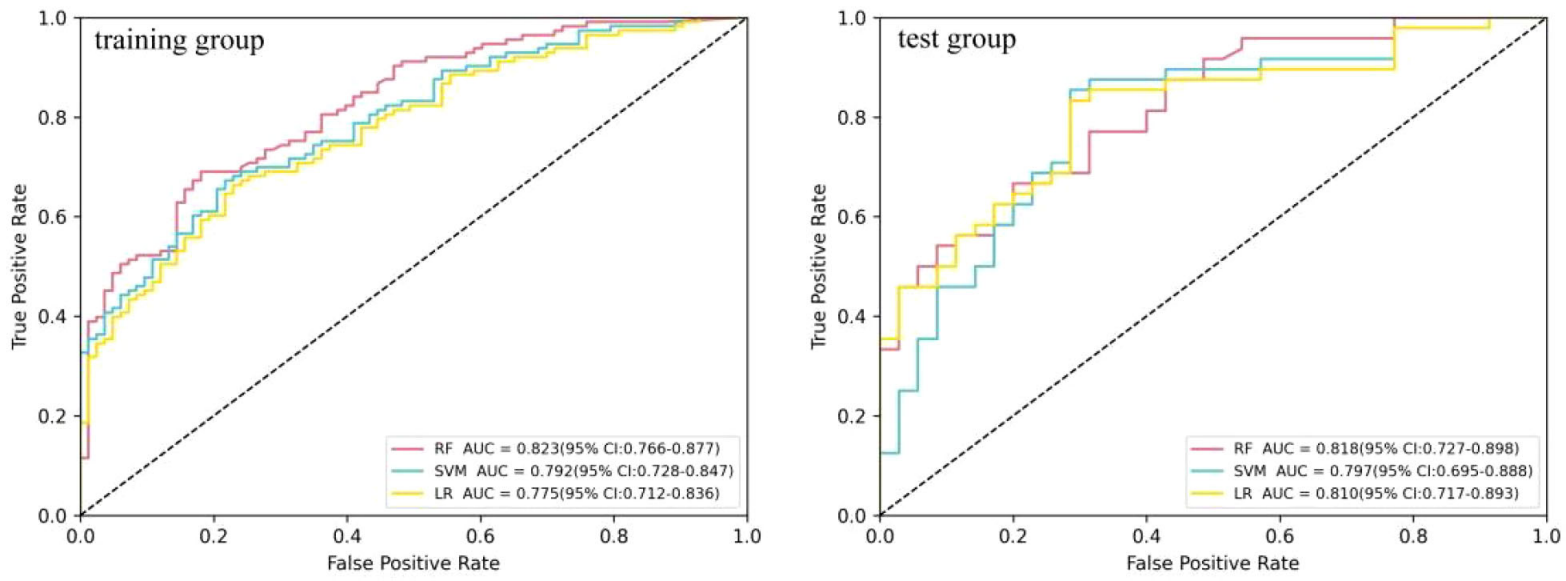
Comparison of the receiver operating characteristic (ROC) diagnostic efficacy of the logistic regression (LR), random forest (RF), and support vector machine (SVM) models in the differential diagnosis of ^18^F-fluorodeoxyglucose (FDG)-avid lymph nodes (LNs).

**Figure 4:**
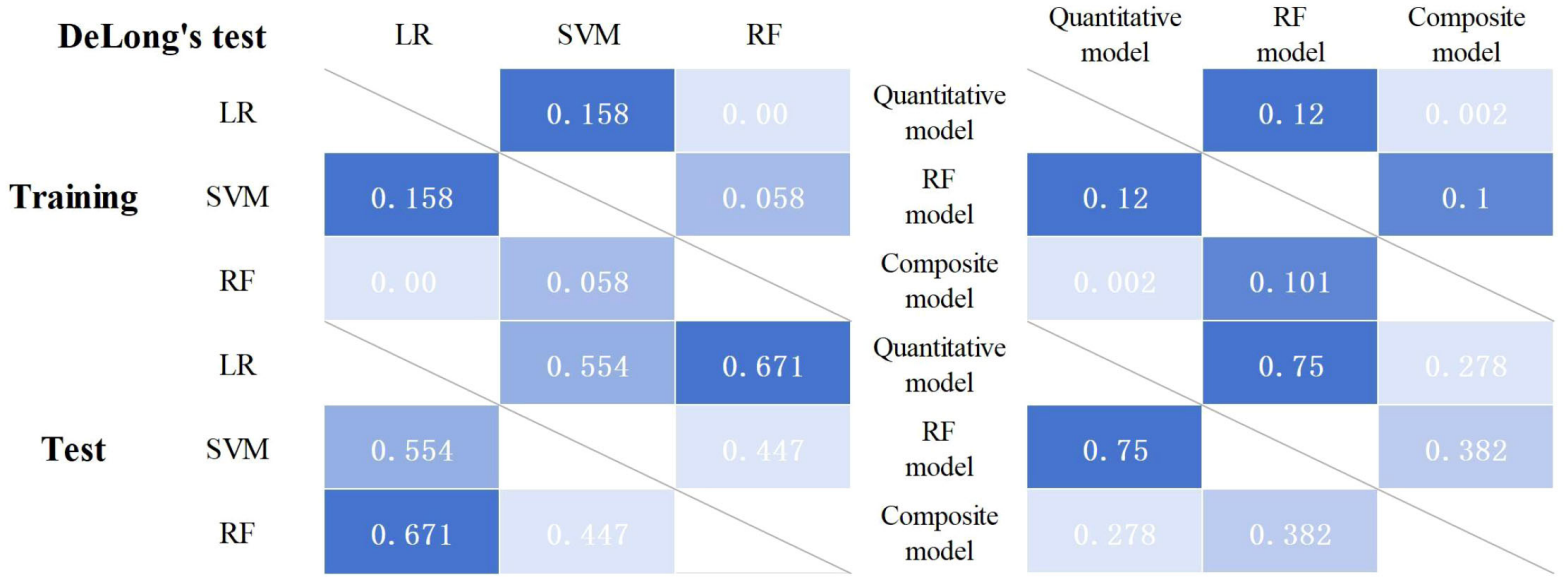
Results of the DeLong’s test for each model.

Three machine learning models (i.e., SVM, RF, and LR) were constructed using six radiomics features. **Figure 3** shows the predictive performance of the three machine learning models for the differential diagnosis of FDG-avid LNs in lung cancer. The ROC curve analysis showed that the RF model had better predictive efficacy in both the training (AUC = 0.823) and test groups (AUC = 0.819). The DeLong’s test showed that, in the training group, there was a statistical difference between LR and RF (*p* < 0.01), but no statistical difference between LR and SVM or RF and SVM (*p* = 0.158 and *p* = 0.058) (**Figure 4**). There were no statistical differences between LR and RF, LR and SVM, or RF and SVM in the test group (*p* = 0.671, 0.554, and 0.447, respectively). **Table 2** shows the predictive efficacy of the three models. For the RF model, the respective values for AUC, sensitivity, specificity, and accuracy were 0.823 (0.766–0.877), 0.69, 0.819, and 0.745 in the training group and 0.818 (0.727–0.898), 0.667, 0.800, and 0.723 in the test group. Therefore, the RF model was included as a predictive model for the PET/CT+*K*_i_ radiomics features.

**Table 2:** Summary of the efficacy of three predictive models in the differential diagnosis of ^18^F-fluorodeoxyglucose (FDG)-avid lymph nodes (LNs).

Model	Group	AUC (95%CI)	Sensitivity	Specificity	Accuracy
RF	Training	0.823 (0.766–0.877)	0.69	0.819	0.745
	Test	0.818 (0.727–0.898)	0.667	0.80	0.723
SVM	Training	0.792 (0.728–0.847)	0.673	0.782	0.719
	Test	0.797 (0.695–0.888)	0.854	0.714	0.795
LR	Training	0.775 (0.712–0.836)	0.664	0.771	0.709
	Test	0.810 (0.717–0.893)	0.833	0.714	0.783

RF, random forest; SVM, support vector machine; LR, logistic regression.

### Composite modeling and effectiveness assessment

**Figure 5:**
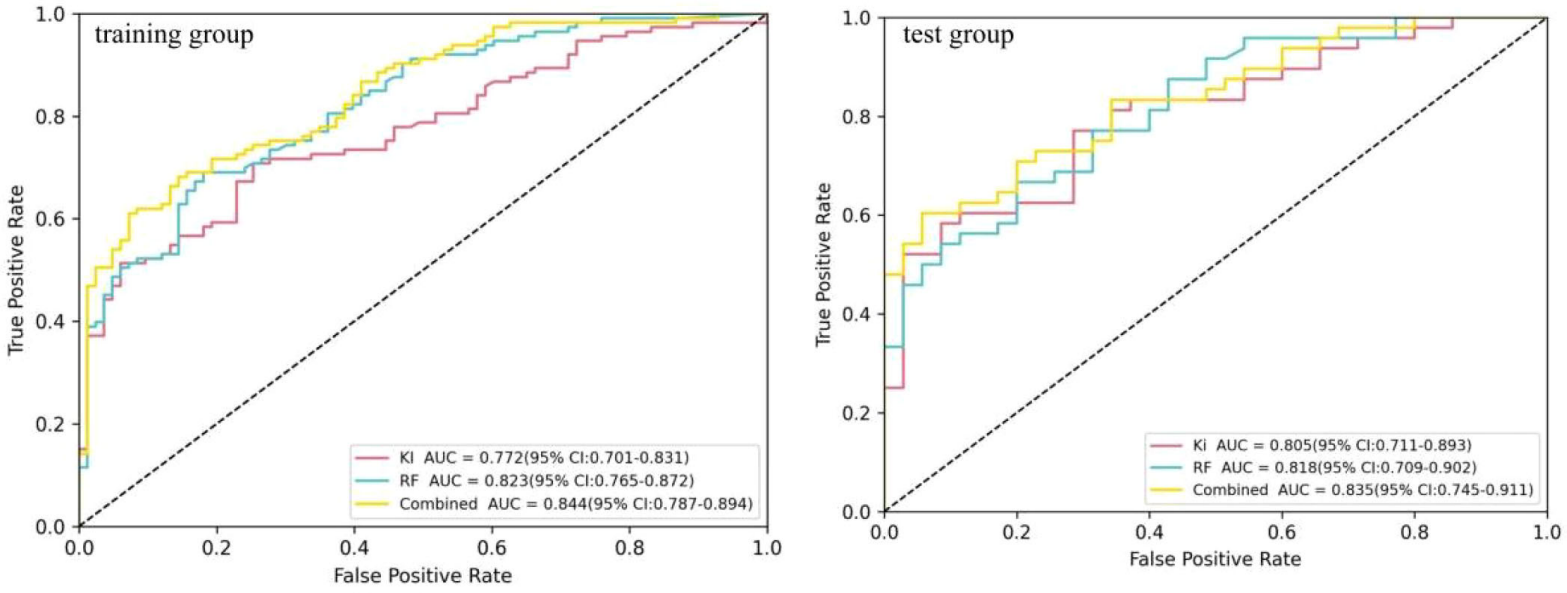
Receiver operating characteristic (ROC) curves for the quantitative model, the random forest (RF) model, and the composite model.

The quantitative model included SUV_max_ and *K*_i_. In the training group, the results of the single-factor logistic regression showed that both the SUV_max_ and *K*_i_ were statistically different in the metastatic and non-metastatic groups (*p* < 0.01, respectively). Multifactor logistic regression analysis showed that *K*_i_ differed significantly between the metastatic and non-metastatic groups (*p* = 0.001), whereas the SUV_max_ did not (*p* = 0.917), as shown in **Table 3**. Therefore, *K*_i_ was included in the quantitative prediction model. Using ROC curve analysis (**Figure 5**), the AUCs of the quantitative model were 0.772 (0.701–0.831) and 0.805 (0.711–0.893) in the training and test groups, respectively.

**Table 3:** Summary of the single-factor and multifactor logistic regression results for the quantitative values.

Quantitative value	Single-factor logistic regression			Multifactor logistic regression		
	OR	95%CI	*p*-value	OR	95%CI	*p*-value
*K*_i_	2.511	1.779–3.765	<0.001	2.464	1.54–4.274	0.001
SUV_max_	1.293	1.165–1.461	<0.001	1.009	0.854–1.191	0.917

A composite prediction model was created based on the RF model and the quantitative model, which was named the PET/CT+*K*_i_+quantitative composite model. According to the ROC curve analysis (**Figure 5**), the AUC, the sensitivity, the specificity, and the accuracy were respectively 0.844 (0.787–0.894), 0.611, 0.928, and 0.745 in the training group and 0.835 (0.745–0.911), 0.604, 0.943, and 0.747 in the test group. The DeLong’s test showed that, in the training group, the quantitative and composite models had a statistical difference (*p* = 0.002), while the quantitative and RF models, as well as the RF and composite models, did not (*p* = 0.120 and *p* = 0.101) (**Figure 4**). There were no statistical differences between the quantitative model and the RF model, the quantitative model and the composite model, or the RF model and the composite model (*p* = 0.750, *p* = 0.278, and *p* = 0.382, respectively). **Table 4** shows the predictive efficacy of the three models.

**Table 4:** Summary of the efficacy of the quantitative model, the random forest (RF) model, and the composite model.

Model	Group	AUC (95%CI)	Sensitivity	Specificity	Accuracy
Quantitative model	Training	0.772 (0.701–0.831)	0.708	0.747	0.724
	Test	0.805 (0.711–0.893)	0.583	0.914	0.723
RF model	Training	0.823 (0.765–0.872)	0.690	0.819	0.745
	Test	0.818 (0.709–0.902)	0.667	0.800	0.723
Composite model	Training	0.844 (0.787–0.894)	0.611	0.928	0.745
	Test	0.835 (0.745–0.911)	0.604	0.943	0.747

**Figure 6:**
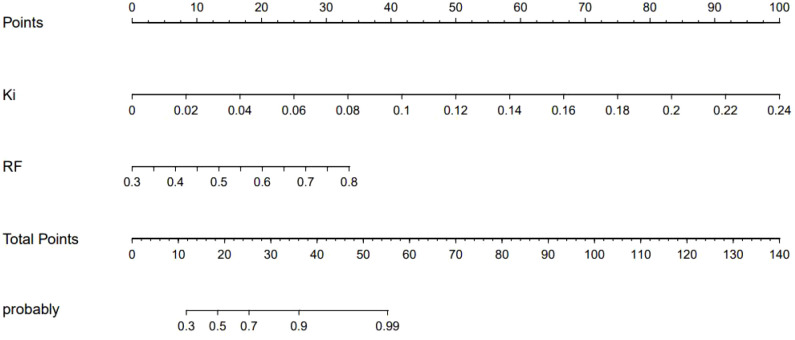
Nomogram of the composite model for predicting the differential diagnosis of ^18^F-fluorodeoxyglucose (FDG)-avid lymph nodes (LNs).

**Figure 7:**
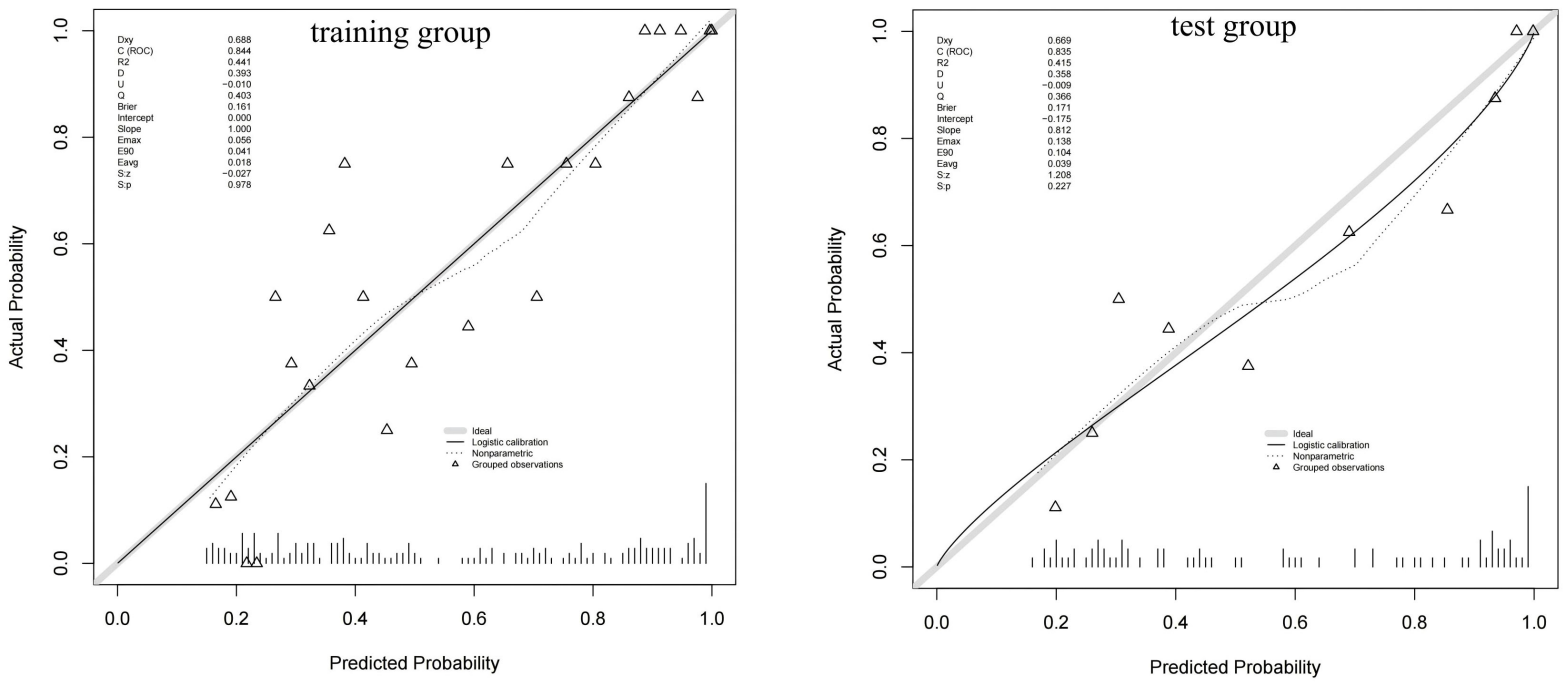
Calibration curves of the composite model for predicting the differential diagnosis of ^18^F-fluorodeoxyglucose (FDG)-avid lymph nodes (LNs).

**Figure 6** shows a nomogram of the composite model, which was constructed based on the machine learning model and the quantitative *K*_i_ prediction scores. Agreement between the predicted and the actual values on the nomogram was evaluated. The results of the Hosmer–Lemeshow goodness-of-fit test for both the training and test groups showed no statistical significance (*p* = 0.978 for the training group and *p* = 0.227 for the test group), indicating that the predictions of the nomogram constructed in this study were unbiased and a perfect fit, as shown in **Figure 7**. The curves demonstrated that the values predicted by the composite model are in close alignment with the actual values.

### Decision curve analysis

**Figure 8:**
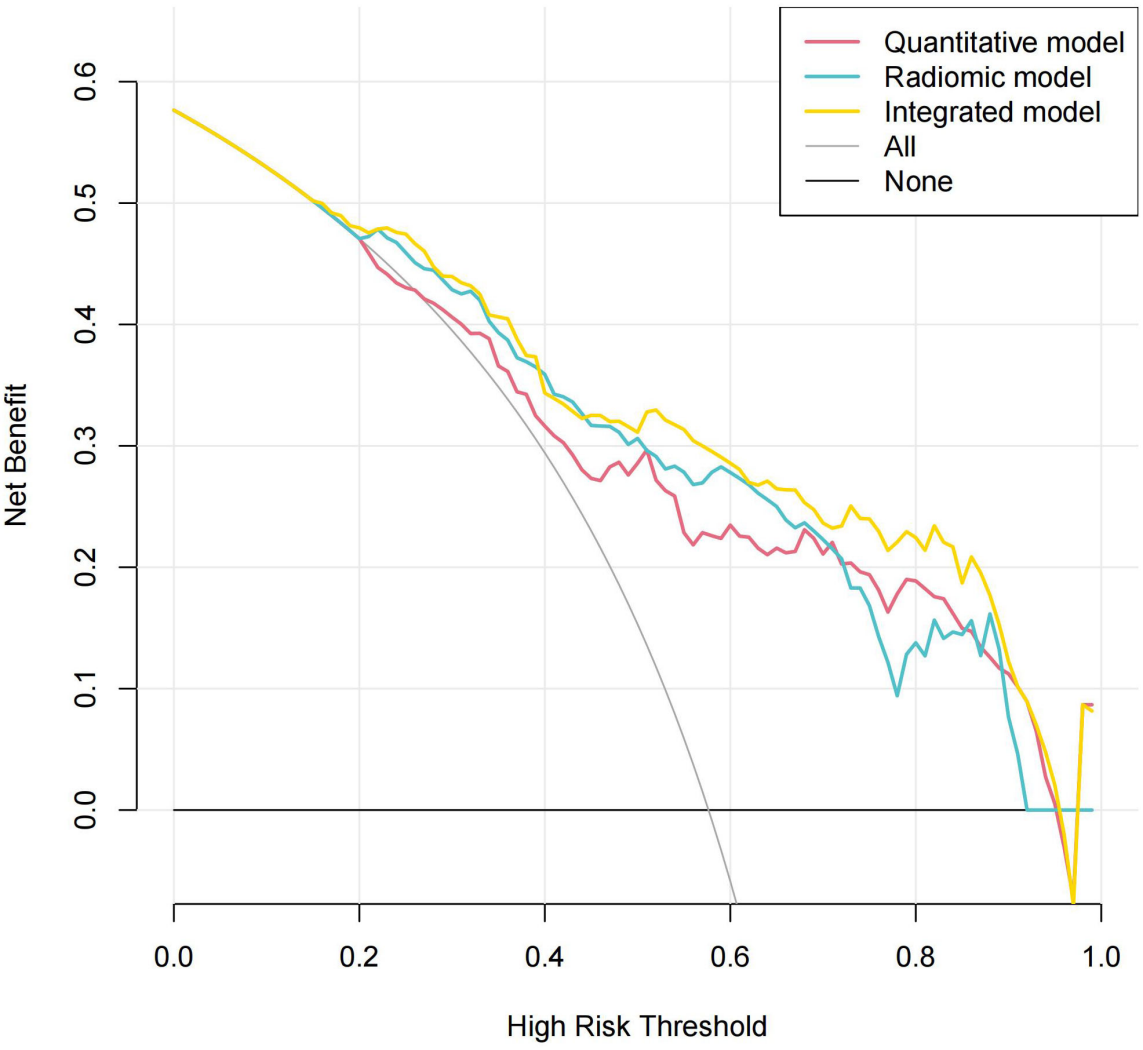
Decision curve analysis of the composite model in predicting the differential diagnosis of ^18^F-fluorodeoxyglucose (FDG)-avid lymph nodes (LNs).

**Figure 8** shows the decision curve analysis of the composite model in predicting the differential diagnosis of FDG-avid LNs in lung cancer. According to the decision curve analysis, the composite model has a better net benefit and clinical value in the differential diagnosis of FDG-avid LNs in lung cancer.

## Discussion

This study investigated the value of a composite model based on the radiomics features from CT, FDG PET, and *K*_i_ images combined with quantitative parameters for predicting the differential diagnosis of FDG-avid LNs in lung cancer. This study concludes that, among the machine learning models, the RF model based on PET/CT+Ki has a high diagnostic value (the training and test group AUCs were 0.823 and 0.818, respectively). In the quantitative model, *K*_i_ had better predictive efficacy (the training and test group AUCs were 0.772 and 0.805, respectively). As a result, our PET/CT+*K*_i_+quantitative composite model had a higher predictive performance (the training and test group AUCs were 0.844 and 0.835, respectively). The clinical decision curves demonstrated that the predicted values of the composite model aligned well with the actual values.

N staging is a key factor in predicting how lung cancer will progress and is vital in developing personalized treatment plans (18). Previous studies by our team on dPET and lung cancer have shown that the dynamic quantitative metabolic parameter *K*_i_ is effective in diagnosing lung cancer and in determining the N stage and EGFR status, particularly in improving the specificity of the differential diagnosis (12–15). In particular, the addition of the dynamic metabolic parameter *K*_i_ reduces the false-positive rate of FDG-avid LNs and improves the accuracy of N staging (12). The combination of dPET and sPET/CT is expected to be an effective tool for the accurate staging of lung cancer.

Radiomics enables the noninvasive identification of solid tumors, as well as the determination of their spatial and temporal consistency, using radiomics features such as pixel density and spatial distribution (19–21). This provides a more complete description of the lesion status. Consequently, radiomics has attracted growing interest in studies related to tumor invasiveness, pathological grading, treatment response, and prognosis prediction. In recent years, there have been reports of studies using radiomics and deep learning in the N staging of lung cancer (19–22). To our knowledge, there are no studies on the use of dynamic imaging for the N staging of lung cancer based on imaging radiomics features.

A previous meta-analysis showed an AUC of 0.90 for predicting LN metastasis in lung cancer using CT and PET radiomics models (23). The CT-based radiomics model demonstrated high sensitivity (0.840), whereas the PET-based radiomics model exhibited a higher specificity (0.860).

Yin et al. (24) concluded that the SVM model based on the FDG PET/CT images is more effective than the RF model in predicting metastatic LNs in lung cancer, with respective AUCs of 0.82 and 0.81. Xie et al. (25) concluded that the combined SUV_max_ and CT radiomics model has better efficacy in the preoperative N staging of lung cancer compared with the SUV_max_ and short diameter, with AUCs of 0.849 and 0.828 for the combined model in the training and test groups, respectively. Our results showed that the diagnostic efficacy of the PET/CT+*K*_i_-based RF model is higher than that of the SVM and LR models. Our results differ from those of previous studies in that we considered the following two factors to be relevant. Firstly, we selected a sample size of FDG-avid LNs on sPET/CT. Secondly, in the current study, we added the imaging group learning feature of dynamic image *K*_i_ to obtain the joint imaging group model PET/CT+*K*_i_.

Yoo et al. (26) concluded that the diagnostic efficacy of the combined FDG PET/CT+clinical information model (AUC = 0.810) is better than that of the physician (AUC = 0.768) or the combined FDG-PET/CT+quantitative values model (AUC = 0.798). Qiao et al. concluded that the PET/CT+tumor location composite model demonstrates high diagnostic efficacy in predicting occult LN metastasis in NSCLC, with a training group AUC of 0.884 (0.826–0.941) and a test group AUC of 0.881 (0.803–0.959) (27). Therefore, it is expected that a comprehensive predictive model combining quantitative values, radiomics features, and clinical information will further improve the accuracy of N staging in lung cancer.

Our previous study showed that *K*_i_ has a higher specificity (0.918 *vs*. 0.388) but a lower sensitivity than SUV_max_ (0.395 *vs*. 0.826) in the differential diagnosis of FDG-avid LNs in lung cancer, which may play a complementary role (15). In a subsequent validation study, it was also concluded that SUV_max_+*K*_i_ could have a higher diagnostic efficacy, with AUC, sensitivity, specificity, and accuracy of 0.907 (0.842–0.951), 84.3%, 94.6%, and 88.89%, respectively (12). Our previous study well established the advantages of *K*_i_ in the N staging of lung cancer, particularly in improving the specificity. In this study, we developed a quantitative prediction model based on *K*_i_.

In this study, our composite model had a higher predictive value for FDG-avid LN metastasis in lung cancer (AUC = 0.844 *vs*. 0.835). The clinical decision curves showed that the composite model had better net benefit and clinical value. In this study, we established a composite model that included a machine learning model based on *K*_i_ images and dynamic metabolic parameters. Therefore, our composite model is expected to be a noninvasive and a reliable imaging method for the accurate N staging of lung cancer, providing clinicians with reliable imaging evidence to guide the development of individualized treatment plans.

This study has several limitations. Firstly, it is based on a single-center image database. A large, multicenter dataset will be required at a later stage to validate the stability and reproducibility of the constructed model. Secondly, based on the results of the preliminary experiments, only three-modality imaging features based on CT, PET, and *K*_i_ were retained in the design of this experiment, and single- or dual-modality CT, PET, or *K*_i_ were not compared. In our subsequent research, we will expand the sample size further and explore comparisons of single-, dual-, and three-modality imaging based on CT, PET, and *K*_i_. Thirdly, due to the limited number of articles related to *K*_i_-based radiomics, particularly those concerning the differential diagnosis of LNs, it was not possible to conduct a horizontal comparison in our discussion. In the future, we intend to conduct more relevant studies based on our institution’s dynamic dataset in order to further explore the clinical value of the radiomics features of *K*_i_ in lung cancer. Finally, the clinical factors in our composite model only included quantitative values (SUV_max_ and *K*_i_). The value of the remaining combined clinical information (e.g., age, gender, and pathology type, among others) will be further explored in later studies.

## Conclusions

A composite model created based on the RF model of PET/CT+*K*_i_ images combined with dynamic quantitative *K*_i_ has high diagnostic efficacy for the differential diagnosis of FDG-avid LNs in lung cancer and has better net benefit and clinical value. The developed composite model is expected to be an effective tool for accurate lung cancer N staging, providing clinicians with reliable imaging evidence to guide the development of individualized treatment plans.

## Data Availability

The raw data supporting the conclusions of this article will be made available by the authors, without undue reservation.
